# Increased Development of Th1, Th17, and Th1.17 Cells Under T1 Polarizing Conditions in Juvenile Idiopathic Arthritis

**DOI:** 10.3389/fimmu.2022.848168

**Published:** 2022-07-04

**Authors:** Anna E. Patrick, Kayla Shoaff, Tashawna Esmond, David M. Patrick, David K. Flaherty, T Brent Graham, Philip S. Crooke, Susan Thompson, Thomas M. Aune

**Affiliations:** ^1^ Department of Pediatrics, Vanderbilt University Medical Center, Nashville, TN, United States; ^2^ Department of Medicine, Vanderbilt University Medical Center, Nashville, TN, United States; ^3^ Department of Veterans Affairs, Nashville, TN, United States; ^4^ Office of Research (OOR) Shared Resources Department, Vanderbilt University Medical Center, Nashville, TN, United States; ^5^ Department of Mathematics, Vanderbilt University, Nashville, TN, United States; ^6^ Department of Pediatrics, Center for Autoimmune Genomics and Etiology, Cincinnati Children’s Hospital Medical Center, University of Cincinnati College of Medicine, Cincinnati, OH, United States; ^7^ Department of Pathology, Microbiology and Immunology, Vanderbilt University Medical Center, Nashville, TN, United States

**Keywords:** juvenile idiopathic arthritis (JIA), interferon gamma (IFNγ), interleukin 17 (IL-17), T cell, T helper cell (Th), Th1.17, Th1 polarization, Th17

## Abstract

In juvenile idiopathic arthritis (JIA) inflammatory T cells and their produced cytokines are drug targets and play a role in disease pathogenesis. Despite their clinical importance, the sources and types of inflammatory T cells involved remain unclear. T cells respond to polarizing factors to initiate types of immunity to fight infections, which include immunity types 1 (T1), 2 (T2), and 3 (T17). Polarizing factors drive CD4^+^ T cells towards T helper (Th) cell subtypes and CD8^+^ T cells towards cytotoxic T cell (Tc) subtypes. T1 and T17 polarization are associated with autoimmunity and production of the cytokines IFNγ and IL-17 respectively. We show that JIA and child healthy control (HC) peripheral blood mononuclear cells are remarkably similar, with the same frequencies of CD4^+^ and CD8^+^ naïve and memory T cell subsets, T cell proliferation, and CD4^+^ and CD8^+^ T cell subsets upon T1, T2, and T17 polarization. Yet, under T1 polarizing conditions JIA cells produced increased IFNγ and inappropriately produced IL-17. Under T17 polarizing conditions JIA T cells produced increased IL-17. Gene expression of IFNγ, IL-17, Tbet, and RORγT by quantitative PCR and RNA sequencing revealed activation of immune responses and inappropriate activation of IL-17 signaling pathways in JIA polarized T1 cells. The polarized JIA T1 cells were comprised of Th and Tc cells, with Th cells producing IFNγ (Th1), IL-17 (Th17), and both IFNγ-IL-17 (Th1.17) and Tc cells producing IFNγ (Tc1). The JIA polarized CD4^+^ T1 cells expressed both Tbet and RORγT, with higher expression of the transcription factors associated with higher frequency of IL-17 producing cells. T1 polarized naïve CD4^+^ cells from JIA also produced more IFNγ and more IL-17 than HC. We show that in JIA T1 polarization inappropriately generates Th1, Th17, and Th1.17 cells. Our data provides a tool for studying the development of heterogeneous inflammatory T cells in JIA under T1 polarizing conditions and for identifying pathogenic immune cells that are important as drug targets and diagnostic markers.

## Introduction

Juvenile idiopathic arthritis (JIA) is the most common autoimmune arthritis in children. Effective therapies for JIA include disease-modifying anti-rheumatic drugs (DMARDs) and biologics that target T cell activation and inflammatory cytokines (1, 2). Most JIA patients attain inactive disease with therapies; however, the likelihood of disease flare is over 50% in the first 2 years after attaining inactive disease (3, 4). Moreover, chronically uncontrolled JIA occurs in half of patients that require multiple DMARDs during treatment (5). Critical needs exist for a personalized approach to current therapies and development of new therapies. Meeting this critical need is hindered by our poor understanding of how inflammatory T cells and cytokines develop in JIA.

In JIA, the clinical and biologic phenotypes are diverse (6). JIA patients with the same clinical subtype can have different biologic phenotypes. Clinical subtypes include more or less involved joints at onset (polyarticular and oligoarticular respectively) and are further subdivided by criteria such as rheumatoid factor (RF), and HLA-B27 positivity and presence of psoriasis in patient or family members (7). The systemic JIA subtype has features of autoinflammatory disease and is distinct (7). The peak JIA incidence is 1-5 years old (8), which is especially true for oligoarticular and polyarticular RF negative subtypes. At this age, the immune system is shifting from more naïve to memory T cells (9, 10). In JIA conflicting evidence exists about whether naïve and memory T cell frequencies are different from healthy children (11–13).

T cells play a major role in adaptive cell immunity. There are at least 3 types of cell-medicated effector immunity, type 1 (T1), type 2 (T2), and type 3 (T17) (14). Both CD4^+^ and CD8^+^ T cells are important in each immunity type and produce cytokines that stimulate the immune system to combat infections. Th1, Th2, and Th17 cells differentiate from naïve CD4^+^ cells in response to T cell receptor stimulation and combinations of polarizing cytokines. These cells selectively express signature cytokines: Th1: IFNγ and TNFα, Th2: IL-4, IL-5, and IL-13, Th17: IL-17 (15–18). Early during Th differentiation, cytokine signaling stimulates phosphorylation of STAT proteins that drives master transcription factor expression. Important STAT proteins for signaling in Th lineages include: Th1: STAT1 and STAT4, Th2: STAT5, Th17: STAT3 (15, 17). Key transcription factors are critical for differentiation of each Th lineage: Th1: Tbet, Th2: GATA3, Th17: RORγT and BATF. During differentiation, each pathway suppresses alternate pathways. CD8^+^ T cells become Tc1, Tc2, and Tc17 cells under parallel respective polarizing conditions and have the same major produced cytokines and master transcription factors, with the addition of Eomes as a Tc1 master transcription factor (14).

T1 and T17 cells and their effector cytokines are inflammatory. In polyarticular and oligoarticular JIA, more Th1 and Th17 cells are present in the synovial fluid of joints with arthritis (19–23). In oligoarticular JIA synovial fluid exhibits type 1 immunity skewing (23). Numbers of effector Th17 cells are elevated in synovial fluid cells in systemic JIA (24). Th17 cells are also found in peripheral blood of children with active JIA (25). The Th17 cell subset exhibits plasticity in autoimmune arthritis and can develop characteristics of Th1 cells (26). In JIA, a Th1-like Th17 cell, Th17.1, is present in synovial fluid and considered pathologic (26). JIA synovial fluid Th17 cells are able to shift to a Th17.1 or Th1 phenotype highlighting the cell plasticity (27). Importantly, in JIA a shift from Th17 cells to Th17.1 cells is reduced in response to treatment with the common JIA therapeutic, etanercept (28). Additionally, abnormal populations of CD8^+^ cells are found in JIA synovial fluid (29). These studies highlight that heterogeneous cell populations are involved in JIA inflammation, yet the origins of these cells are unclear.

We find that JIA peripheral blood mononuclear cells (PBMCs) undergo abnormal T cell polarization and inappropriately produce inflammatory cytokines, IFNγ and IL-17, in short-term T cell cultures that model T cell differentiation and cytokine production (14, 30, 31). With a focus on prepubescent children, we hypothesize that JIA precursor T cells are predisposed to heightened inflammation under T cell polarizing conditions.

## Materials and Methods

### Patients and Samples

JIA PBMCs from Vanderbilt Monroe Children’s Hospital and the Cincinnati Children’s Hospital Pediatric Rheumatology Tissue Repository and child healthy control (HC) PBMCs from the Cincinnati Children’s Hospital Medical Center genomic control cohort were obtained with IRB approved protocols. Isolated PBMC were stored in fetal bovine serum with 10% DMSO in liquid nitrogen.

### T Cell Cultures and Cytokines

PBMCs were cultured under T1, T2, and T17 polarizing conditions in a well-defined tissue culture model for T cell differentiation and cytokine secretion (14, 30–32). The T17 polarizing conditions include anti-IFNγ. Cultured cells were in RPMI 1640 (Gibco) supplemented with 10% fetal bovine serum (Atlanta Biologicals), L-glutamine (Gibco), and penicillin-streptomycin (Gibco) with 5% air CO_2_. PBMCs at 100,000 cells/well in a 96-well plate or 500,000 cells/well in a 24-well plate were stimulated on anti-CD3 (10µg/mL) precoated plates with anti-CD28 (1µg/mL) and stimuli for T cell polarization. Stimuli were T1: IL-12 (10ng/mL), T2: IL-4 (10ng/mL), T17: IL-6 (50ng/mL), IL-1β (20ng/mL), TGF-β1 (2ng/mL), IL-23 (20ng/mL), IL-21 (100ng/mL), anti-IFNγ (10µg/mL). Antibodies were purchased from Biolegend and cytokines were purchased from BD Biosciences and R&D Systems. After 5 days for T1 and T2 cultures or 7 days for T17 cultures, media containing polarization factors was washed away and cells were re-stimulated on new anti-CD3 coated plates with media that did not contain polarization factors. After 2 days, culture fluids and cells were harvested for analysis. Culture fluids were analyzed for cytokines by ELISA to detect human IFNγ (BD Biosciences), TNFα (BD Biosciences), IL-17a (ThermoFisher), IL-5 (BD Biosciences), and IL-13 (ThermoFisher). T cell cultures were performed in triplicate and ELISA results averaged.

Naïve CD4^+^ cells were isolated from PBMCs using the EasySep™ Human CD4+ T Cell Isolation Kit (StemCell) using immunomagnetic negative selection according to the manufacturer’s instructions. Naïve CD4 T cell cultures had 200,000 cells/well in a 96-well plate that were stimulated on anti-CD3 (10µg/mL) precoated plates with anti-CD28 (1µg/mL) and IL-2 (100U/mL, PeproTech). In some samples T1 polarization factors were added.

### Flow Cytometry

PBMC were analyzed with LIVE/DEAD™ Fixable Violet Dead stain, anti-CD3 APC-H7, anti-CD8a PE, anti-CD4 perCP-cy5.5, anti-CD197 AF647, and anti-CD45RA FITC with standard flow cytometry protocols. Gating was performed with fluorescent minus one (FMO) control for CD197^+^ and CD45RA^+^ populations. Experiments were performed on a 3-Laser BD LSRFortessa instrument maintained by the Vanderbilt University Medical Center Flow Cytometry Shared Resource.

T1 cell cultures were restimulated on day 5 and then analyzed on day 7 after being treated with PMA (30ng/mL), ionomycin (1µg/mL), and GolgiStop (1µL/mL) for 6 hours. T1 cells were analyzed with Fixable Zombie NIR, anti-CD3 AF700, anti-CD4 BV510, anti-CD8 BV711, anti-IFNγ PEDazzle594, anti-IL-17 APC, anti-Tbet PE-Cy7, and anti-RORγT PE with standard flow cytometry protocols. Gating was performed with FMO control for IFNγ^+^ and IL-17^+^ populations. Experiments were performed on a Cytek Aurora instrument.

Naïve CD4^+^ cells were isolated and primary cells were analyzed or T1 polarized cells were analyzed. Primary cells were placed on anti-CD3 coated plates with added anti-CD28 and IL-2 for 16 hours, then treated with Cell Stimulation Cocktail (plus protein transport inhibitors) for 5 hours, and analyzed by flow cytometry. Naïve CD4^+^ cells were isolated and underwent T1 polarization on anti-CD3 coated plates with added anti-CD28, IL-2, and IL-12 for 5 days, then were treated with Cell Stimulation Cocktail (plus protein transport inhibitors) for 5 hours, and were analyzed by flow cytometry. Primary cells and T1 cells were analyzed with Fixable Zombie NIR, anti-CD3 AF700, anti-CD4 BV510, anti-CD8 BV711, anti-IFNγ PEDazzle594, and anti-IL-17 APC with standard flow cytometry protocols. Experiments were performed on a Cytek Aurora instrument.

Analysis was performed using FlowJo software. Reagents were purchased as antibodies from BD Pharmingen, Biolegend, and Life Technologies and reagents from BD Biosciences and ThermoFisher.

### Cell Proliferation

PBMCs were labeled with carboxyfluorescein succinimidyl ester (CFSE)(10µM) using the CellTrace CFSE Cell Proliferation Kit (ThermoFisher, C34554). Cells were then stimulated with immobilized anti-CD3 and anti-CD28 (1µg/mL). On day 3, harvested cells were analyzed by flow cytometry for live/dead cells, anti-CD3 APC-H7, anti-CD8a BV711, and anti-CD4 APC. Purchased reagents are from Life Technologies, BD Pharmingen, BD Biosciences, and Biolegend. Experiments were performed on a 3-Laser BD LSRFortessa instrument. Analysis was performed using FlowJo software Proliferation Modeling.

### RNA Analysis

RNA was collected from T1 and T2 cell cultures polarized for 5 days using TRIreagent and complementary DNA synthesized using SuperScript First Strand Synthesis (ThermoFisher). Quantitative real time PCR measured expressed RNAs using SYBR green master mix (Applied Biosystems). RNA levels were standardized relative to the housekeeping gene GAPDH. Patient samples containing definite outliers (ROUT with Q=0.1% by GraphPad Prism software) were removed. Patient samples with both T1 and T2 available were included for analysis.

For RNA sequencing, RNA was collected by TRIreagent followed by DNase digestion. Library preparation was performed with the Illumina Tru-Seq Stranded RNA kit. RNA sequencing was performed with an Illumina HiSeq2500 instrument by the Vanderbilt Technologies for Advanced Genomics (VANTAGE) core facilities consecutively on all samples. 100bp paired end reads were generated. Average sequencing depth of all samples was 43 million mapped reads ± 13.5 million (standard deviation). FASTQ files were processed using DESeq2 to identify differentially expressed genes (33). Data are available in NCBI’s Gene Expression Omnibus (34) with GEO series accession number GSE185193. GO Enrichment analysis for overrepresented biological processes was performed using the Gene Ontogeny Resource (35–37).

### Statistical Analysis

Applied statistical tests were Welch’s t-test for comparison of 2 and Welch Anova for comparison of more than 2 samples. P < 0.05 was considered significant.

## Results

### Clinical Characteristics of JIAs and Healthy Controls

We performed studies in prepubescent children with JIA and age-, gender-, and race-matched controls (HC) (**Table 1**). JIA patients were an average of 62 months old, ANA+, and mostly female, consistent with known clinical characteristics. JIA subtypes were defined using International League Against Rheumatism (ILAR) criteria (38). Clinical characteristics were collected at the time of PBMC collection (**Table 1**). Overall, JIA patients had 0-1 active joints with arthritis and were on methotrexate and/or biologic medications including etanercept, adalimumab, infliximab, and abatacept. Five JIA PBMCs were collected within 1 month of diagnosis and before initiation of systemic medications other than non-steroidal anti-inflammatories (NSAIDs). This group had a higher number of active joints at time of biosample collection, with an average of 6 active joints.

**Table 1:** Demographic and clinical characteristics of JIA and child healthy control groups.

	HC	JIA
Number of cases	20	20
Age in months (range)	62 (36-93)	62 (24-94)
Gender, female, n (%)	16 (80%)	16 (80%)
Ethnicity, Caucasian, n (%)	19 (95%)	19 (95%)
ANA positive, n (%) *		19 (95%)
JIA subtypes, n (%)		
Polyarticular RF-		17 (85%)
Polyarticular RF+		1 (5%)
Oligoarticular		2 (10%)
**Characteristics at Time of PBMC Collection**		
Active joints, n (%)		
0-1		12 (60%)
2-4		3 (15%)
>4		5 (25%)
Disease duration <1 month, n (%)		5 (25%)
Time since diagnosis in months (range)		17.5 (0-70)
Physician global assessment score (range)		2 (0-9)
Current medications, n (%)		
Methotrexate only		5 (25%)
Biologic only		3 (15%)
Methotrexate + Biologic		6 (30%)
Prednisone only		1 (5%)
None or NSAID		5 (25%)

*1ANA unknown, child healthy controls (HC).

### Frequencies of Naïve and Memory T cells in JIA and HC

**Figure 1:**
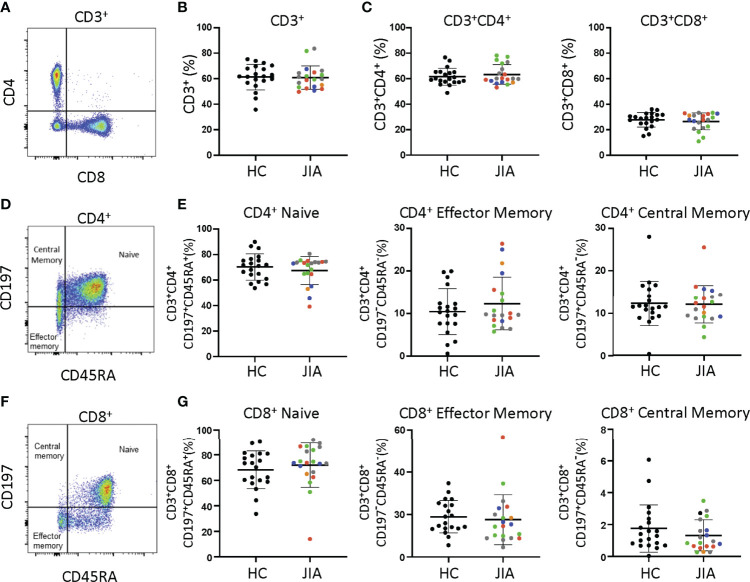
T cell subsets from child healthy control (HC) and JIA are the same. HC and JIA PBMCs analyzed with flow cytometry for CD3^+^, CD3^+^CD4^+^, and CD3^+^CD8^+^ cells and for naïve (CD197^+^CD45RA^+^), central memory (CD197^+^CD45RA^-^), and effector memory (CD197^-^CD45RA^-^) cell subsets. JIA are colored based on current use of methotrexate (green), biologic (blue), methotrexate and biologic (gray), prednisone (orange), and none or NSAID (red). **(A)** Representative flow cytometry plot of CD3^+^ cells. **(B)** Analysis of CD3^+^ cell frequency. **(C)** Analysis of CD3^+^CD4^+^ and CD3^+^CD8^+^ cell frequencies. **(D)** Representative flow cytometry plot of CD3^+^CD4^+^ cells. **(E)** Analysis of naïve, effector memory, and central memory frequency of CD3^+^CD4^+^ cells. **(F)** Representative flow cytometry plot of CD3^+^CD8^+^ cells. **(G)** Analysis of naïve, effector memory, and central memory frequency of CD3^+^CD8^+^ cells. HC (N=20) and JIA (N=20). Shown is mean with standard deviation. Analysis by Welch’s t-test. No significant differences.

Differences between JIA and HC peripheral blood mononuclear cells (PBMCs) were investigated by analyzing T cell subsets and naïve and memory phenotypes. JIA and HC PBMCs had the same frequencies of CD3^+^, CD3^+^CD4^+^, and CD3^+^CD8^+^ cells (**Figures 1A-C**). On average, 60% were CD3^+^ cells, of which approximately 63% were CD4^+^ and 28% were CD8^+^. Memory and naïve cell phenotypes were identified by expression of CD197 (CCR7) and CD45RA with naïve cells: CD197^+^CD45RA^+^, central memory cells: CD197^+^CD45RA^-^, and effector memory cells: CD197^-^CD45RA^-^. Pediatric (JIA and HC) CD3^+^CD4^+^ cells were an average of 69% naïve and 23% combined central and effector memory cells (**Figures 1D, E**). Four JIAs had naive CD3^+^CD4^+^ cells below the standard deviation and effector memory CD3^+^CD4^+^ cells above the standard deviation. These 4 JIA patients were all polyarticular RF- JIA with no common features shown by age in months (range 23-83), active joints (range 0->9), months since JIA diagnosis (range 0-54), and physician global assessment score (range 0-5). Pediatric CD3^+^CD8^+^ cells were an average of 70% naïve and 12% combined effector and memory cells (**Figures 1F, G**). In pediatric patients, ratios of naïve to memory CD3^+^CD4^+^ and CD3^+^CD8^+^ cells were 3 and 5.8 respectively. Importantly, no differences in memory and naïve cell phenotypes were identified between JIA and HC. Groups were color coded based on current use of methotrexate, biologic, methotrexate and biologic, prednisone, or none or NSAID therapy to assess the role of therapies on cell frequencies. There was no clustering of patient samples based on current therapy (**Figure 1**).

### T Cell Proliferation in JIA and HC

JIA and HC T cell proliferation was assessed by labeling PBMCs with the fluorescent dye, CFSE. Labeled cells were stimulated with anti-CD3 and anti-CD28 and proliferation measured on day 3 (**Figure S1A**). The proliferation index (PI), which is the number of divisions per dividing cell, for JIA and HC CD3^+^, CD3^+^CD4^+^, and CD3^+^CD8^+^ cells were not different (**Figure S1B**). On average, PIs for CD3^+^, CD3^+^CD4^+^, and CD3^+^CD8^+^ cells were 1.4, 1.4, and 1.5 (**Figure S1B**). Importantly, no differences in PIs were identified between JIA and HC.

### Increased IFNγ and IL-17 Production by JIA T Cell Cultures

**Figure 2:**
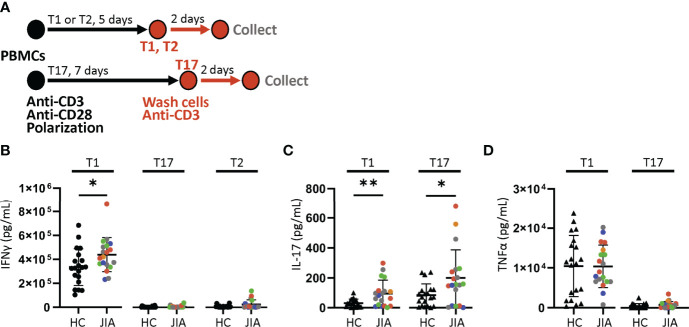
Increased production of IFNγ and IL-17 from JIA polarized T cells. T1, T2, and T17 cells were polarized *in vitro* from child healthy control (HC) and JIA PBMCs. JIA are colored based on current use of methotrexate (green), biologic (blue), methotrexate and biologic (gray), prednisone (orange), and none or NSAID (red). **(A)** Schematic of polarization showing PBMCs treated with anti-CD3, anti-CD28 and polarization conditions for 5 days for T1 and T2 cells or 7 days for T17 cells. The polarization media is then removed, cells are washed, new media without polarization factors added, and activated with anti-CD3. After 2 days, the produced cytokines were analyzed by ELISA. **(B)** IFNγ produced by T1, T17, and T2 cells for HC and JIA with new diagnosis JIA denoted (red circle). **(C)** IL-17 produced by T1 and T17 cells for HC and JIA with new diagnosis JIA denoted (red circle). IL-17 was below detection for T2 cells. **(D)** TNFα produced by T1 and T17 cells for HC and JIA with new diagnosis JIA denoted (red circle). TNFα was below detection for T2 cells. HC (N=20) and JIA (N=19). Shown is mean with standard deviation. Analysis by Welch’s t-test *p < 0.05, **p < 0.01.

We determined if JIA T cells, which were largely naïve, developed inflammatory cytokine profiles with T cell polarization. We used short-term T cell cultures to model T cell polarization and cytokine production (30, 31). JIA and HC PBMCs underwent T1, T2, and T17 polarization, activation, and were then collected for analysis (**Figure 2A**). In T1 cultures, JIA exhibited increased production of IFNγ (**Figure 2B**). While in T2 cultures of HC IFNγ was completely suppressed, IFNγ was detectable in several JIA T2 cultures. JIA T1 and T17 cultures had increased production of IL-17 (**Figure 2C**). Notably, IL-17 production in JIA T1 cultures was similar or even greater than its production in HC T17 cultures. No differences were detected in TNFα levels (**Figure 2D**). Granulocyte macrophage colony-stimulating factor (GM-CSF) was analyzed in a subset of T1 polarized cultures and no differences were detected. In T2 cultures IL-17 and TNFα production were below the limits of detection. To ensure T2 cultures polarized appropriately, IL-13 and IL-5 were measured and showed that both cytokines were expressed and were not different between JIA and HC (**Figures S2A, B**). To determine if the 5 newly diagnosed JIA, who were receiving no therapy or NSAIDs, had a skewed pattern of inflammatory cytokine production, these patients were identified in our T cell culture analysis (**Figure 2**, red circles). The newly diagnosed JIA were not different than total JIA for T1, T2, and T17 culture production of IFNγ, IL-17, and TNFα. Groups were color coded based on current use of methotrexate, biologic, methotrexate and biologic, prednisone, or none or NSAID therapy to assess the role of therapies on cytokine production. There was no clustering of patient samples based on therapy (**Figures 2B-D**). A separate JIA subject had longitudinal biosamples at two timepoints that were separated by 5 months. Both biosamples were obtained while the patient was on methotrexate. The longitudinal JIA samples had T1 polarized cells with heightened production of IFNγ and IL-17 compared to HC (**Figure S3**).

To determine if differences in cytokine production were due to T cell subset frequencies, HC and JIA T1, T2, and T17 CD3^+^CD4^+^ and CD3^+^CD8^+^ cells were analyzed by flow cytometry. We found that the subsets are distributed similar between JIA and HC cultures (**Figures S4A, B**).

### Increased Expression of mRNAs Encoding IFNγ, IL-17, Tbet and RORγT in JIA T1 Cells

**Figure 3:**
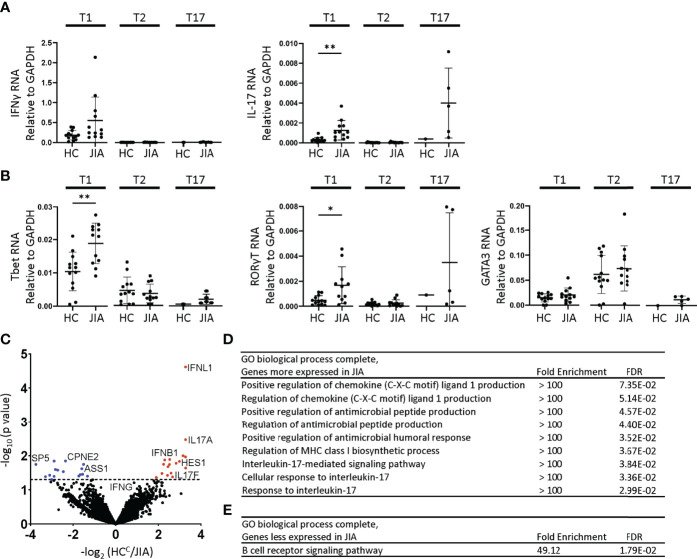
Altered gene expression and T1 biologic pathway activation in JIA. RNA expression in child healthy control (HC) and JIA T1, T2, and T17 cells using RT-PCR for **(A)** IFNγ, IL-17 and **(B)** Tbet, RORγT, and GATA3. JIA T1 and T2 (N=12), HC T1 and T2 (N=13). JIA T17 (N=5) and HC T17 (N=1). Analysis by Welch’s t-test with *p<0.05, **p<0.01 for T1 and T2 comparisons. **(C)** Volcano plot of differentially expressed genes in HC (N=3) and JIA (N=3) T1 cultures by RNA sequencing analyzed by DESeq2. Genes overexpressed or underexpressed in JIA with p-value <0.05 (above dashed line). PANTHER Overrepresentation Test from the GO Ontology database for genes **(D)** overexpressed or **(E)** underexpressed in JIA.

T1, T2, and T17 polarization in Th and Tc cells involve similar STAT signaling, master transcription factor induction, and signature cytokine expression. We assessed gene expression levels in JIA and HC T1, T2, and T17 cells by quantitative real time qPCR. The cells used for this experiment were generated in parallel with the cytokine production data in **Figure 2**. The inflammatory cytokine IFNγ was expressed higher in T1 than T2 and T17 cells (**Figure 3A**). The inflammatory cytokine IL-17 was expressed higher in T1 and T17 cells than T2 cells (**Figure 3A**). IFNγ expression was magnitudes higher than IL-17. Importantly, IL-17 was expressed at significantly higher levels in JIA T1 cells than in HC (**Figure 3A**). IFNγ exhibited a trend of higher expression in JIA T1 cells than in HC. IL-17 exhibited a trend of higher expression in JIA T17 cells than in HC. Master transcription factors Tbet, RORγT, and GATA3 are important in T1, T17, and T2 polarization, respectively. We found Tbet was more highly expressed in T1 cells and GATA3 was more highly expressed in T2 cells (**Figure 3B**). RORγT was more highly expressed in T1 cells and T17 cells. Importantly, both Tbet and RORγT were expressed significantly higher in JIA T1 cells than in HC (**Figure 3B**). In STAT signaling, STAT4 and STAT1 increase Tbet and are important in T1 polarization and STAT3 increases RORγT and is important in T17 polarization. STAT4, STAT1, and STAT3 gene expression levels were not different between JIA and HC in T1 and T2 cells (**Figure S5**).

Biological pathway differences in JIA and HC T1 cells were determined by RNA sequencing and pathways analysis. RNA from 3 HC and 3 JIA T1 cells were analyzed for differential gene expression using DeSeq2. The 3 JIA patients were selected based on showing production of IFNγ and IL-17 in T1 polarized cultures, RNA availability, and having an age-matched HC with RNA available. The RNA was collected from a T1 polarized culture prepared in parallel with samples from **Figure 2**. The demographic and clinical characteristics of the 3 HC and JIA samples were compared (**Table S1**). Genes with p-value less than 0.05 were identified and found 17 genes with increased and 16 genes with decreased expression in JIA (**Figure 3C**) (**Table S2**). The most overexpressed JIA T1 protein coding genes were IFNL1 and IL17A. The JIA genes with increased expression were analyzed for enriched biological processes using a PANTHER overrepresentation test. The identified biologic processes included important immune pathways and the IL-17 mediated signaling pathway (**Figure 3D**). The JIA genes with decreased expression were analyzed for enriched biologic processes and found enrichment in the B cell receptor signaling pathway (**Figure 3E**). RNA sequencing data identified pathways associated with IL-17 as highly dysregulated in JIA T1 cells.

### JIA T1 Th Cells Were IFNγ, IL-17, and Dual IFNγ-IL-17 Producers and Tc Cells Were IFNγ Producers

**Figure 4:**
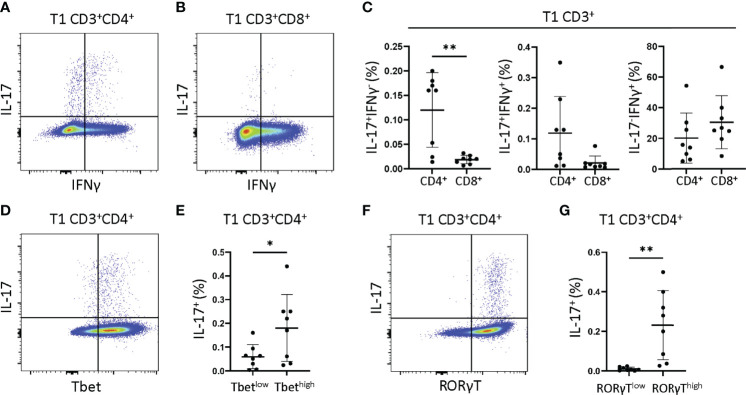
JIA T1 cells have single IFNγ^+^ and IL-17^+^ and dual IFNγ^+^IL-17^+^ cells that express Tbet and RORγT. JIA T1 cells were assessed with flow cytometry to identify IFNγ^+^ and IL-17^+^ cell subsets. Representative flow cytometry plot from JIA T1 cells showing IFNγ and IL-17 positive cells and gating for **(A)** CD3^+^CD4^+^ cells and **(B)** CD3^+^CD8^+^ cells. **(C)** JIA T1 CD3^+^ cell frequency of CD4^+^ and CD8^+^ cells that are IFNγ^-^IL-17^+^, IFNγ^+^IL-17^+^, and IFNγ^+^IL-17^-^. **(D)** Representative flow cytometry plot from JIA T1 CD3^+^CD4^+^ cells showing IL-17 and Tbet. **(E)** JIA T1 CD3^+^CD4^+^ cells showing frequency of IL-17 positive cells in Tbet^low^ and Tbet^high^ subsets. **(F)** Representative flow cytometry plot from JIA T1 CD3^+^CD4^+^ cells showing IL-17 and RORγT. **(G)** JIA T1 CD3^+^CD4^+^ cells showing frequency of IL-17 positive cells in RORγT^low^ and RORγT^high^ subsets. Shown is mean with standard deviation. Analysis by Welch’s t-test with *p<0.05, **p<0.01.

We next asked if JIA T1 cell IFNγ and IL-17 were derived from single or dual cytokine producing cells, and if these cells were Th cells (CD3^+^CD4^+^) or Tc cells (CD3^+^CD8^+^). JIA T1 cultures from 8 patients were analyzed by flow cytometry for frequency of cells producing IFNγ, IL-17 or both IFNγ-IL-17. Representative flow cytometry diagrams of JIA T1 cells that are CD3^+^CD4^+^ and CD3^+^CD8^+^ showed cell production of IFNγ and IL-17 (**Figures 4A, B**). IL-17 was produced in CD3^+^CD4^+^ and not in CD3^+^CD8^+^ JIA T1 cells. IL-17 was produced in CD3^+^CD4^+^ cells that made only IL-17 and cells that made both IFNγ-IL-17. IFNγ was produced in both CD3^+^CD4^+^ and CD3^+^CD8^+^ JIA T1 cells. The frequency of IFNγ^-^IL-17^+^, IFNγ^+^IL-17^+^, and IFNγ^+^IL-17^-^ cells were determined in the CD3^+^CD4^+^ and CD3^+^CD8^+^ JIA T1 cell subsets. A significantly higher frequency of IFNγ^-^IL-17^+^ cells was present in CD3^+^CD4^+^ cells (**Figure 4C**). A trend of higher frequency of IFNγ^+^IL-17^+^ cells was present in CD3^+^CD4^+^ cells (**Figure 4C**). We next asked if the expression of the master transcription factors Tbet and RORγT were associated with IL-17 production in JIA T1 CD3^+^CD4^+^ cells. A representative flow cytometry diagram of JIA T1 CD3^+^CD4^+^ cells showed expression of IL-17 in cells that are Tbet^high^ and Tbet^low^ (**Figure 4D**). The frequency of IL-17 positive cells in JIA T1 CD3^+^CD4^+^Tbet^high^ cells was higher than in CD3^+^CD4^+^Tbet^low^ cells (**Figure 4E**). A representative flow cytometry diagram of JIA T1 CD3^+^CD4^+^ cells showed expression of IL-17 in cells that were RORγT^high^ and RORγT^low^ (**Figure 4F**). The frequency of IL-17 positive cells in JIA T1 CD3^+^CD4^+^RORγT^high^ cells was significantly higher than in CD3^+^CD4^+^RORγT^low^ cells (**Figure 4G**).

We then compared whether the production of cytokines in T1 polarized cultures by ELISA correlates with production of cytokines in T1 polarized cells measured in CD3^+^CD4^+^ cells by flow cytometry. We found that JIA T1 polarized culture measures were significantly correlated for IFNγ (**Figure S6A**) and trended towards significance for IL-17 (**Figure S6B**). We then analyzed RNA from T1 polarized cells generated during the flow cytometry experiment, finding that the JIA T1 polarized cells expressed high levels of the IFNγ, IL-17, Tbet, and RORγT genes (**Figure S7**).

### JIA Naïve CD4^+^ Cells that Underwent T1 Polarization Become both IFNγ and IL-17 Producers

**Figure 5:**
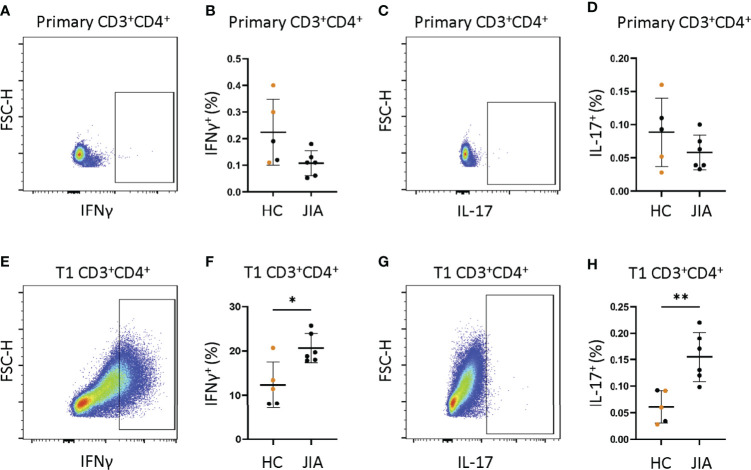
JIA naïve CD4^+^ cells become IFNγ and IL-17 producers under T1 polarizing conditions. Naïve CD4^+^ cells JIA (n=6) and HC (n=3 adult (orange) and 2 child healthy controls (black)) were analyzed by flow cytometry for production of IFNγ and IL-17 in primary cells and T1 polarized cells. **(A)** Representative flow cytometry plot from JIA primary naïve CD4^+^ cells showing IFNγ positive cells. **(B)** Primary naïve CD4^+^ cells frequency of IFNγ positive cells in HC and JIA. **(C)** Representative flow cytometry plot from JIA primary naïve CD4^+^ cells showing IL-17 positive cells. **(D)** Primary naïve CD4^+^ cells frequency of IL-17 positive cells in HC and JIA. **(E)** Representative flow cytometry plot from JIA T1 polarized naïve CD4^+^ cells showing IFNγ positive cells. **(F)** T1 polarized naïve CD4^+^ cells frequency of IFNγ positive cells in HC and JIA. **(G)** Representative flow cytometry plot from JIA T1 polarized naïve CD4^+^ cells showing IL-17 positive cells. **(H)** T1 polarized naïve CD4^+^ cells frequency of IL-17 positive cells in HC and JIA. Shown is mean with standard deviation. Analysis by Welch’s t-test with *p<0.05, **p<0.01.

We asked if isolated naïve CD4^+^ JIA cells generated more IFNγ and IL-17 from primary cells and T1 polarized cells than naïve CD4^+^ HC cells. Naïve CD4^+^ cells from 6 JIA patients and 5 HCs (3 adult and 2 pediatric) were analyzed by flow cytometry for frequency of CD3^+^CD4^+^ cells producing IFNγ and IL-17. A representative flow cytometry diagram of JIA primary CD3^+^CD4^+^ cells showed low expression of IFNγ (**Figure 5A**). The frequency of IFNγ positive cells in JIA and HC primary CD3^+^CD4^+^ cells was not different (**Figure 5B**). A representative flow cytometry diagram of JIA primary CD3^+^CD4^+^ cells showed low expression of IL-17 (**Figure 5C**). The frequency of IL-17 positive cells in JIA and HC primary CD3^+^CD4^+^ cells was not different (**Figure 5D**). A representative flow cytometry diagram of JIA T1 CD3^+^CD4^+^ cells showed high expression of IFNγ (**Figure 5E**). The frequency of IFNγ positive cells in JIA T1 CD3^+^CD4^+^ cells was significantly higher than HC CD3^+^CD4^+^ cells (**Figure 5F**). A representative flow cytometry diagram of JIA T1 CD3^+^CD4^+^ cells showed higher expression of IL-17 (**Figure 5G**). The frequency of IL-17 positive cells in JIA T1 CD3^+^CD4^+^ cells was significantly higher than HC CD3^+^CD4^+^ cells (**Figure 5H**). Only JIA T1 CD3^+^CD4^+^ cells exhibited an increase in IL-17 frequency among all isolated naïve CD4^+^ cells studied. Importantly, the frequencies of IFNγ and IL-17 positive JIA CD4^+^ cells generated from PBMCs (**Figure 4C**) and naïve CD4^+^ cells (**Figures 5F-H**) by T1 polarization was similar.

## Discussion

In this study, we demonstrate that T cells from JIA patients develop an inflammatory cytokine profile in response to T1 and T17 polarization. On initial assessment, JIA and HC T cells have the same naïve and memory T cell profiles and proliferative capacity. After T1 polarization, JIA cells express increased IFNγ and IL-17 and increased IL-17, Tbet and RORγT mRNA. This is surprising since T1 polarization should effectively suppress the T17 polarization that is associated with RORγT and IL-17. This contrasts with T1 polarization in HC cells where both RORγT and IL-17 are suppressed. JIA T1 polarization leads to single IFNγ, single IL-17, and dual IFNγ-IL-17 producing cells that are CD3^+^CD4^+^ and are Th1, Th17, and Th1.17 cells respectively. JIA T1 polarization of naïve CD4+ cells generates high IFNγ and IL-17 producing cells, indicating that JIA naïve CD4^+^ cells have an increased drive to produce inflammatory cytokines. Under T17 polarizing conditions, JIA T cells produce increased IL-17. Under T2 differentiation conditions, JIA T cells do not express IFNγ or IL-17. Our work demonstrates that T cells in JIA have a pro-inflammatory profile using *in vitro* T1 and T17 cell polarization, and that naïve JIA CD4+ cells have the capacity to inappropriately become IL-17 producers under T1 polarizing conditions.

A striking finding is the similarity between JIA and HC T cells. Similarly, polyarticular RF- JIA patients and controls do not exhibit different memory and naïve T cell phenotypes (13). Despite the similarities in lymphocytes, a subset of hyperresponsive JIA CD4^+^ T cells did respond more strongly to IFNγ (13). In our data we analyzed 4 JIA samples with a lower percentage of naïve CD3^+^CD4^+^ cells and a higher percentage of effector memory CD3^+^CD4^+^ cells. These 4 samples did not cluster during the assessment of IFNγ or IL-17 production in T1 and T17 polarization conditions. Our findings are not related to the percent of input naïve or memory cells. Additionally, JIA and HC T cell proliferative capacity is similar. A limitation in this study is that a small population of dividing cells or an antigen specific population may not be identified. Another limitation is that we did not have longitudinal samples for all JIA patients in this study. However, we have a JIA patient with longitudinal samples and this patient exhibited the inflammatory T cell phenotype at both timepoints, suggesting the phenotype is stable. Importantly, we minimize age dependent immune effects by focusing on a prepubescent population in both JIA and HC.

JIA synovial fluid has an increase in Th1 cells, Th17 cells, abnormal CD8^+^ cells, and associated cytokines for oligoarticular, polyarticular, and enthesitis-arthritis subtypes (19–21, 23, 29, 39). This parallels our T cell culture findings that T1 polarization produces Th1, Th17, and Th1.17 cells. An unexpected result from our T1 polarization is the increase in IL-17. In our transcriptome analysis of T1 polarized cells, IL-17 associated pathways are enriched. In our JIA T1 culture cells, the expression of high Tbet and RORγT correlates with IL-17 production, supporting that the master transcription factors contribute to production of IL-17. Further transcriptome studies from sorted cell populations will better define the pathways activated in the IFNγ, IL-17, and dual IFNγ-IL-17 producing cell populations.

In our studies, JIA T17 polarization produces more IL-17 and suppresses IFNγ, which is expected for a Th17 cell. The T17 polarization conditions include multiple cytokines and antibodies, including anti-IFNγ. One limitation is that we do not know the importance of each individual component to our described phenotype. Further studies focusing on T17 polarization will be required to delineate the mechanism of our result. T2 polarization of PBMCs does not generate IFNγ and IL-17, suggesting that Th1, Th17, and Th1.17 cells are generated during the process of T1 polarization rather than already existing in PBMCs. This is supported by studies of JIA naïve CD4^+^ cells that showed primary cells do not produce IFNγ and IL-17 and after T1 polarization higher levels of IFNγ and IL-17 are produced compared to HCs.

Dual IFNγ and IL-17 producing CD4^+^ Th cells called Th17.1, non-classic Th1, and ex-Th17 cells are identified in various autoimmune conditions, often at sites of inflammation (27, 40–43). JIA synovial fluid contains Th17.1 cells (26, 27). Th17.1 cells begin as Th17 cells that produce IFNγ in response to IL-12, then dual producing cells stop producing IL-17 and become sole IFNγ producers (26). In our study, we call the CD4^+^ JIA dual IFNγ and IL-17 producing cells Th1.17 cells. The JIA T1 polarization has more IL-17, Tbet and RORγT at the gene expression level. Normally, IL-12 drives T1 polarization to Th1 cells that increases Tbet and IFNγ and suppresses RORγT and IL-17 expression (44). IL-12 also shifts the Th17.1 cells to become sole IFNγ producers (26). Our culture conditions result in different findings, with IL-12 driving generation of dual-producing cells and RORγT and IL-17 expression. How Th1.17 cells are made during T1 polarization is unknown. There are several possibilities that are not mutually exclusive. One hypothesis is that JIA cells are resistant to IFNγ-induced suppression of Th17 pathways, resulting in IFNγ and IL-17 dual producers. A second possibility is that a small proportion of memory T cells expands to produce these cells. A third hypothesis is that JIA naïve T cells have an abnormal response to IL-12 resulting in dual activation of Tbet and RORγT driven pathways. In our studies, JIA naïve CD4^+^ cells produce IFNγ and IL-17 in response to T1 polarization. This supports that an abnormal response to IL-12 and T1 polarization contributes to the abnormal inflammatory cells. Additional studies are necessary to fully differentiate between these hypotheses and identify mechanistic drivers of Th1.17 cell production in JIA. Importantly, in JIA the inflammatory T1 and T17 phenotype is not present in T2 polarization suggesting counterregulatory pathways could prevent the inflammatory cells.

Biologic heterogeneity is present in JIA, and biologic phenotypes do not always clearly align with clinical phenotypes (6). During our studies, this biologic heterogeneity is exhibited by the observation that JIA PBMCs have a range of IFNγ and IL-17 production. How heterogeneity in JIA develops and whether it is due to genetics or environment or a combination of both is poorly understood. For example, a JIA patient with a monogenetic loss of function mutation in GATA3 exhibits a similar phenotype to the patients herein, with an increase in IFNγ and IL17 in T1 polarized cultures and an increase in IL17 in T17 polarized cultures (31). The molecular basis of this finding is due to loss of GATA3 function. Another study shows that rare variants are present in JIA and that these rare variants associate with immune pathways (45). Additionally, a JIA genome wide association study shows that the identified loci are over-represented in the Th17 cell differentiation pathway (46). An important question is, in JIA do rare mutations and genetic changes in the T1 and T17 polarization pathways contribute to development of IFNγ and IL-17 producing cells? Are JIA T cells genetically pre-programmed towards heightened inflammation? Determining whether JIA T cells have an innate tendency to become IFNγ and IL-17 producing cells, and how genetic mutations might contribute to this phenotype, may open new avenues for understanding disease onset pathogenesis and developing personalized approaches to JIA medication choices and diagnostics.

## Data Availability Statement

The datasets presented in this study can be found in online repositories. The names of the repository/repositories and accession number(s) can be found in the article/**Supplementary Material**.

## Ethics Statement

The studies involving human participants were reviewed and approved by Vanderbilt University Medical Center Institutional Review Board and Cincinnati Children’s Institutional Review Board. Written informed consent to participate in this study was provided by the participants’ legal guardian/next of kin.

## Author Contributions

AP and TA contributed to conception and design of the study. AP, KS, TE, DP, DF performed experiments and statistical analysis. PC performed bioinformatics and statistical analysis. AP, TG, and ST obtained patient biosamples and clinical data. AP and TA wrote the manuscript and prepared figures. All authors reviewed the manuscript. All authors contributed to the article and approved the submitted version.

## Funding

This work was supported by the National Institutes of Health [R01AI044924 to TA], [P30AR070549 and P01AR048929 to ST], [K08HL153789 and IK2BX005376 to DP], [K12HD087023 to Research Scholar AP]. The VUMC Flow Cytometry Shared Resource was supported by the National Institutes of Health [P30CA68485 to the Vanderbilt Ingram Cancer Center] and [DK058404 to the Vanderbilt Digestive Disease Research Center]. The Cincinnati Genomic Control Cohort was supported by the Cincinnati Children’s Research Foundation. This study was supported by the Ann M. Duffer Family Foundation. This study was supported by the Childhood Arthritis and Rheumatology Research Alliance and the Arthritis Foundation (CARRA-AF) [Fellows Small Grant to AP].

## Conflict of Interest

The authors declare that the research was conducted in the absence of any commercial or financial relationships that could be construed as a potential conflict of interest.

## Publisher’s Note

All claims expressed in this article are solely those of the authors and do not necessarily represent those of their affiliated organizations, or those of the publisher, the editors and the reviewers. Any product that may be evaluated in this article, or claim that may be made by its manufacturer, is not guaranteed or endorsed by the publisher.
