# Supplementary material for: Increased Development of Th1, Th17, and Th1.17 Cells Under T1 Polarizing Conditions in Juvenile Idiopathic Arthritis
Source: Front Immunol. 2022 Jul 4;13:848168. doi: 10.3389/fimmu.2022.848168 (PMC9290377; doi:10.3389/fimmu.2022.848168)
Supplement: Supplementary file 1 [file DataSheet_1.docx]

Supplementary Material

**
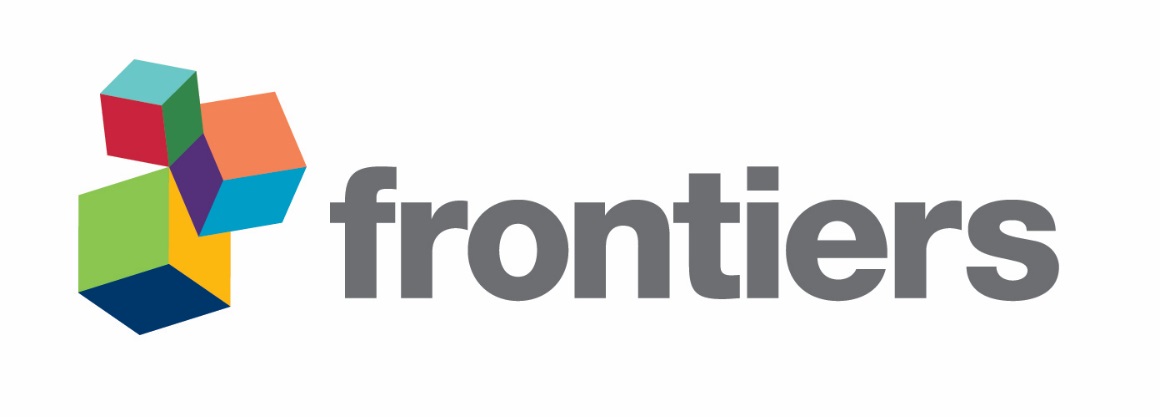
**

**SUPPLEMENTAL FIGURE 1.** T cell proliferation from child healthy control (HC) and JIA are the same. CFSE based T cell proliferation assay of HC and JIA PBMCs measured by flow cytometry on day 3. Proliferation index (PI) is the number of divisions per dividing cell. **(A)** Representative CD3^+^ cell flow cytometry plot of CFSE-stained cells. **(B)** Analysis of PI for CD3^+^, CD3^+^CD4^+^, and CD3^+^CD8^+^ cells. HC (N=14) and JIA (N=11). Shown is mean with standard deviation. Analysis by Welch’s t-test. No significant differences.

**
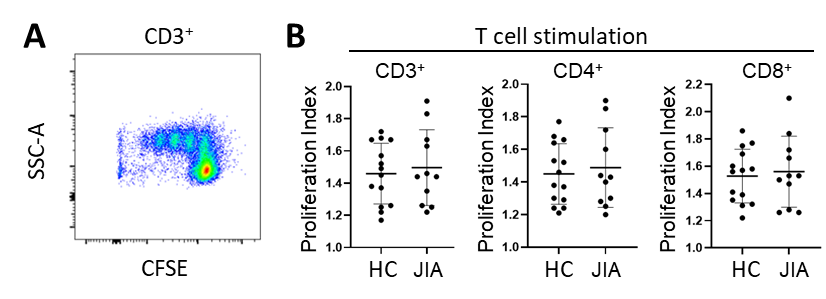
**

**SUPPLEMENTAL FIGURE 2.** Production of IL-5 and IL-13 from child healthy control (HC) and JIA *in vitro* polarized T2 cells. **(A)** IL-5 produced by T2 cells for HC and JIA. **(B)** IL-13 produced by T2 cells for HC and JIA. Shown is mean with standard deviation. Analysis by Welch’s t-test. No significant differences.


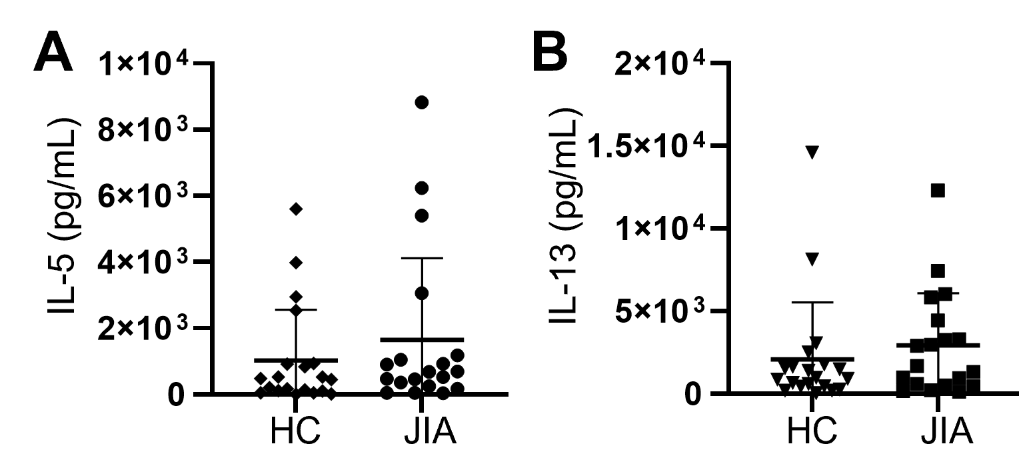


**SUPPLEMENTAL FIGURE 3.** T1 polarized cells from a single JIA patient at two timepoints separated by 5 months compared to two age matched child healthy controls (HC) showing IFNγ and IL-17 production by ELISA. The HC samples are nearly overlapping.

**
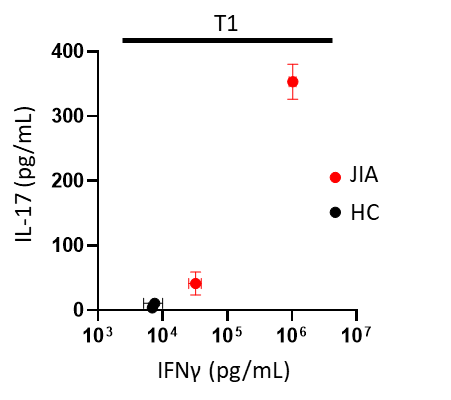
**

**SUPPLEMENTAL FIGURE 4.** T1, T2, and T17 cell subset frequencies vary and are the same between child healthy control (HC) and JIA. Polarized T1, T2, and T17 cells were analyzed by flow cytometry for CD3^+^CD4^+^ and CD3^+^CD8^+^ T cell subsets. **(A)** Analysis of CD3^+^CD4^+^ cell frequency and **(B)** CD3^+^CD8^+^ cell frequency in T1, T2, and T17 cells. T1 HC (N=11), T1 JIA (N=11), T2 HC (N=11), T2 JIA (N=10), T17 HC (N=11), T17 JIA (N=10). Shown is mean with standard deviation. Analysis by Welch’s t-test. No significant differences.

**
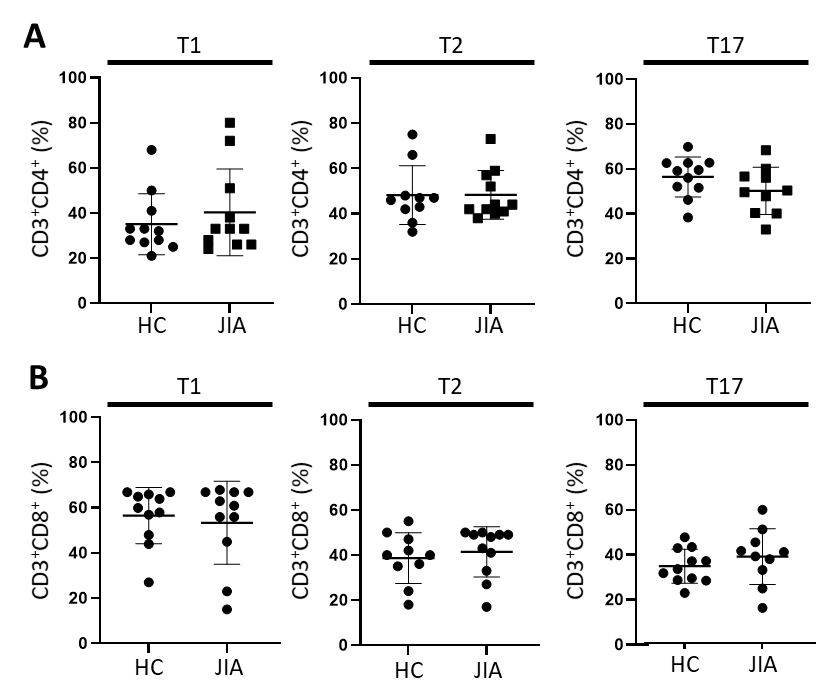
**

**SUPPLEMENTAL FIGURE 5.** STAT gene expression in child healthy control (HC) and JIA T1 and T2 cells. Gene expression of the STAT transcription factors STAT4, STAT1, and STAT3 were examined in HC and JIA polarized T1 and T2 cells using quantitative RT-PCR. HC T1 and T2 (N=13), JIA T1 (N=11), and JIA T2 (N=12). Gene expression is relative to GAPDH. Shown is mean and standard deviation. Analysis by Welch’s t-test. No significant differences.

**
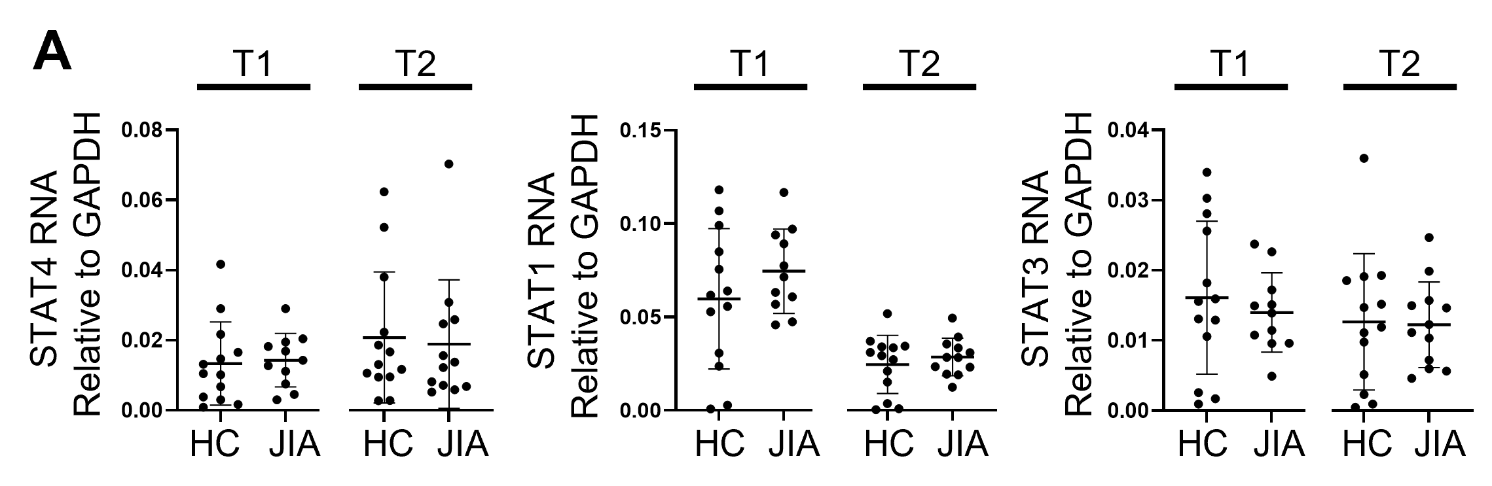
**

**SUPPLEMENTAL FIGURE 6.** Comparison of JIA T1 polarized culture production of cytokines by ELISA and flow cytometry. The 8 JIA samples with both data available were analyzed using a simple linear regression. **(A)** IFNγ production measured by ELISA compared to flow cytometry frequency of CD3^+^CD4^+^IFNγ^+^ cells. **(B)** IL-17 production measured by ELISA compared to flow cytometry frequency of CD3^+^CD4^+^IL-17^+^ cells.

**
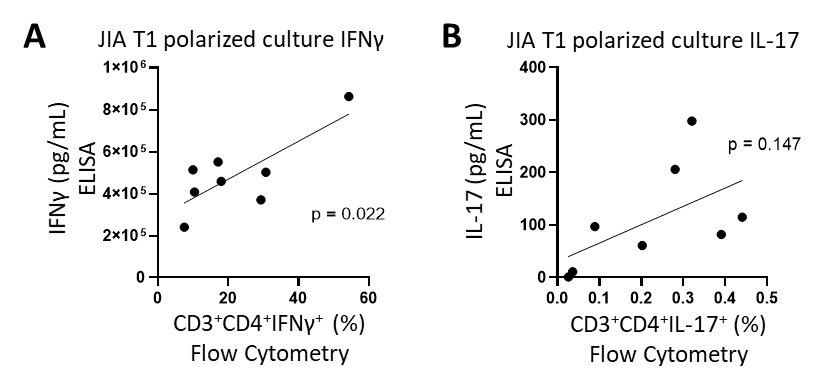
**

**SUPPLEMENTAL FIGURE 7.** Gene expression in JIA T1 polarized cells analyzed for flow cytometry (JIA^flow^) compared to T1 polarized JIA (JIA) and child healthy control (HC) T1 cells from Figure 3. Gene expression of IFNγ, IL-17, Tbet, and RORγT were examined in JIA^flow^ using quantitative RT-PCR. HC T1 (N=13), JIA T1 (N=11), and JIA^flow^ T1 (N=7). Gene expression is relative to GAPDH. Shown is mean and standard deviation.


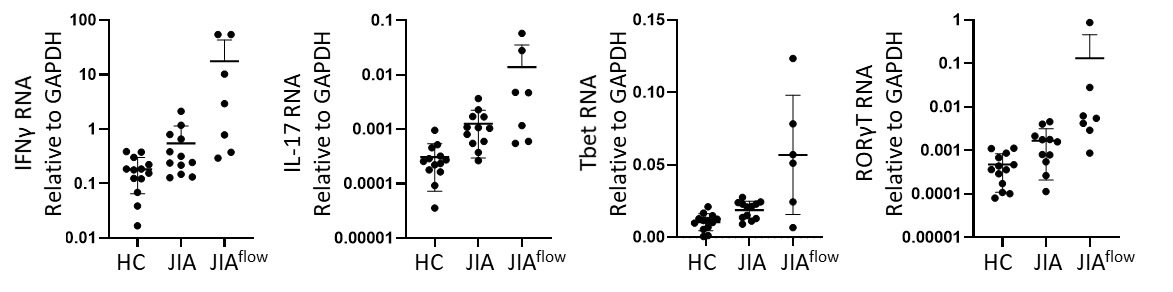


**SUPPLEMENTAL TABLE S1.** Demographic and clinical characteristics of 3 JIA and 3 child healthy control groups used for RNA sequencing.

|  | HC | JIA |
| --- | --- | --- |
| Number of cases | 3 | 3 |
| Age in months (range) | 60 (48-79) | 61 (48-84) |
| Gender, female, n (%) | 1 (33%) | 1 (33%) |
| Ethnicity, Caucasian, n (%) | 3 (100%) | 3 (100%) |
| ANA positive, n (%) * |  | 3 (100%) |
| JIA subtypes, n (%) |  |  |
| Polyarticular RF- |  | 3 (100%) |
|  |  |  |
| **Characteristics at Time of PBMC Collection** | | |
| Active joints, n (%) |  |  |
| 0-1 |  | 1 (33%) |
| >4 |  | 2 (67%) |
| Disease duration <1 month, n (%) |  | 0 (0%) |
| Time since diagnosis in months (range) |  | 8 (2-15) |
| Physician global assessment score (range) |  | 3 (1-5) |
| Current medications, n (%) |  |  |
| Methotrexate only |  | 1 (33%) |
| Methotrexate + Biologic |  | 2 (67%) |

**SUPPLEMENTAL TABLE S2.** T1 cell differentially expressed genes between JIA and child healthy control with p <0.05.

|  | **baseMean** | **log2FoldChange** | **lfcSE** | **stat** | **pvalue** | **Ensemble** |
| --- | --- | --- | --- | --- | --- | --- |
| LINC00887 | 229.5036228 | -3.282064262 | 1.444277997 | -2.27246019 | 0.023058729 | ENSG00000214145 |
| IFNL1 | 258.1045835 | -3.280737223 | 0.776968794 | -4.22248261 | 2.42E-05 | ENSG00000182393 |
| IL17A | 270.8269026 | -3.277124939 | 1.119003182 | -2.92861092 | 0.003404803 | ENSG00000112115 |
| TSKS | 235.5305558 | -3.275720858 | 1.286906837 | -2.54542190 | 0.010914577 | ENSG00000126467 |
| CTD.3222D19.12 | 294.3359079 | -3.158557878 | 1.227074285 | -2.57405596 | 0.010051401 | ENSG00000269399 |
| HES1 | 126.7289088 | -2.987802108 | 1.224955559 | -2.43911061 | 0.014723461 | ENSG00000114315 |
| RP5.890E16.2 | 229.4133056 | -2.833468025 | 1.184252234 | -2.39262206 | 0.016728463 | ENSG00000263412 |
| IL17F | 2317.845574 | -2.66606902 | 1.309882749 | -2.03534936 | 0.041815732 | ENSG00000112116 |
| TMEM213 | 189.6759105 | -2.595901796 | 1.214562309 | -2.13731463 | 0.032572409 | ENSG00000214128 |
| IFNB1 | 161.8574342 | -2.532044582 | 1.018380609 | -2.48634406 | 0.012906312 | ENSG00000171855 |
| SPSB2 | 512.4596369 | -2.51991147 | 1.066840919 | -2.36203113 | 0.018175116 | ENSG00000111671 |
| ALDH4A1 | 317.4531246 | -2.452131272 | 1.063467843 | -2.30578788 | 0.021122487 | ENSG00000159423 |
| PLAT | 264.691779 | -2.428523342 | 1.169882525 | -2.07586940 | 0.037906031 | ENSG00000104368 |
| RP4.575N6.4 | 314.7985737 | -2.298054783 | 0.928821924 | -2.47416078 | 0.013354958 | ENSG00000225938 |
| CBLN3 | 284.0859812 | -2.244566683 | 0.947984473 | -2.36772515 | 0.017897826 | ENSG00000139899 |
| OLIG3 | 235.0267266 | -2.170276142 | 1.019424638 | -2.12892259 | 0.033260665 | ENSG00000177468 |
| LGALS17A | 121.6447874 | -1.906670276 | 0.94451033 | -2.01868652 | 0.043519812 | ENSG00000226025 |
| RPL23AP7 | 681.8760017 | 1.326339363 | 0.645494049 | 2.054766215 | 0.039901591 | ENSG00000240356 |
| IGFBP4 | 1703.92069 | 1.50895516 | 0.638139996 | 2.364614611 | 0.018048842 | ENSG00000141753 |
| CD79A | 1196.922598 | 1.550079696 | 0.735851558 | 2.106511399 | 0.035159954 | ENSG00000105369 |
| ASS1 | 5514.66332 | 1.591156362 | 0.705868508 | 2.254182393 | 0.024184696 | ENSG00000130707 |
| RAB38 | 466.1593356 | 1.659537425 | 0.790881514 | 2.098338872 | 0.035875221 | ENSG00000123892 |
| PDLIM1 | 723.6751717 | 1.73225893 | 0.841364041 | 2.0588697 | 0.039506722 | ENSG00000107438 |
| CPNE2 | 545.8580394 | 2.35572233 | 0.962119066 | 2.448472764 | 0.014346328 | ENSG00000140848 |
| IGHG4 | 89.29080387 | 2.457653534 | 1.128559093 | 2.177691491 | 0.029429013 | ENSG00000211892 |
| IGFBP2 | 3038.714142 | 2.720820675 | 1.226558216 | 2.218256451 | 0.026537352 | ENSG00000115457 |
| RP11.253E3.1 | 115.599896 | 2.828023209 | 1.266370837 | 2.23317146 | 0.02553764 | ENSG00000234589 |
| IGHG1 | 1448.149994 | 2.889952643 | 1.181303481 | 2.446409995 | 0.014428682 | ENSG00000211896 |
| JCHAIN | 112.0140031 | 2.896349233 | 1.414098409 | 2.048194959 | 0.0405409 | ENSG00000132465 |
| IGHA2 | 70.74929883 | 3.009949306 | 1.535003057 | 1.960875122 | 0.049893592 | ENSG00000211890 |
| RPL23AP4 | 97.7935494 | 3.098787818 | 1.489313048 | 2.080682649 | 0.037462963 | ENSG00000212932 |
| IGHGP | 107.814981 | 3.299373358 | 1.616838141 | 2.040633057 | 0.041287314 | ENSG00000253755 |
| SP5 | 99.92999701 | 3.744806407 | 1.583113431 | 2.365469418 | 0.01800723 | ENSG00000204335 |
